# Interleukin-33 produced by M2 macrophages and other immune cells contributes to Th2 immune reaction of IgG4-related disease

**DOI:** 10.1038/srep42413

**Published:** 2017-02-13

**Authors:** Sachiko Furukawa, Masafumi Moriyama, Kensuke Miyake, Hitoshi Nakashima, Akihiko Tanaka, Takashi Maehara, Mana Iizuka-Koga, Hiroto Tsuboi, Jun-Nosuke Hayashida, Noriko Ishiguro, Masaki Yamauchi, Takayuki Sumida, Seiji Nakamura

**Affiliations:** 1 Section of Oral and Maxillofacial Oncology, Division of Maxillofacial Diagnostic and Surgical Sciences, Faculty of Dental Science, Kyushu University, Fukuoka, Japan; 2 OBT Research Center, Faculty of Dental Science, Kyushu University, Japan; 3 Division of Innate Immunity, Department of Microbiology and Immunology, Institute of Medical Science, University of Tokyo, Tokyo, Japan; 4 Division of Nephrology and Rheumatology, Department of Internal Medicine, Faculty of Medicine, Fukuoka University, Fukuoka, Japan; 5 Department of Internal Medicine, Faculty of Medicine, University of Tsukuba, Tsukuba, Japan; 6 Department of Microbiology and Immunology, Keio University School of Medicine, Tokyo, Japan

## Abstract

IgG4-related disease (IgG4-RD) is characterized by elevated serum IgG4 and marked infiltration of IgG4-positive cells in multiple organs. Interleukin-33 (IL-33) is a recently described cytokine that is secreted by damaged epithelial cells, macrophages, and dendritic cells, and potently activates helper T type 2 (Th2) immune responses, which have been suggested to play a major role in IgG4 production of IgG4-RD. Here, we assessed the expression of IL-33 and related molecules in the salivary glands (SGs) of patients with IgG4-RD versus that in patients with Sjögren’s syndrome (SS) and controls. Expression of IL-33 and its receptor (ST2) was strongly detected around ectopic germinal centers (GCs) in the SGs from patients with IgG4-RD, whereas IL-33 was expressed only in epithelial cells in patients with SS and controls. Moreover, IL-33 and CD68^+^/CD163^+^ macrophages were mainly distributed around ectopic GCs in patients with IgG4-RD. Double immunofluorescence staining showed that IL-33 expression co-localized with CD68^+^/CD163^+^ macrophages. Finally, mRNA expression levels of IL-33 showed a positive correlation to those of Th2 cytokines (IL-4 and IL-13) in patients with IgG4-RD. Our data suggest that IL-33 produced by M2 macrophages might contribute to the pathogenesis of IgG4-RD via aberrant activation of Th2 immune responses.

Immunoglobulin (Ig) G4-related disease (IgG4-RD) is a recently recognized inflammatory disorder that comprises many autoimmune diseases such as type I autoimmune pancreatitis (AIP) and retroperitoneal fibrosis. IgG4-RD is characterized by elevated serum IgG4, as well as sclerosing, severe fibrosis, and IgG4-positive cell infiltration in affected organs^1^, which include the lacrimal gland (LG), submandibular gland (SMG), kidney, bile ducts, pancreas, prostate and mammary glands. Affected tissues can be diagnosed using comprehensive IgG4-related disease or organ-specific diagnostic criteria^2^.

Mikulicz’s disease (MD) and Küttner tumor (KT) affect the oral maxillofacial region, presenting with changes in facial appearance, such as persistent swollen of upper eyelids, parotid gland, and submandibular glands. Owing to histopathological similarities, MD is considered a subtype of Sjögren’s syndrome (SS)^3^; however, Yamamoto *et al*. reported that patients with MD and IgG4-RD share many clinical and pathological characteristics^4^. Thus, MD has been regarded as an IgG4-RD, specifically referred to as IgG4-related dacryoadenitis and sialoadenitis (IgG4-DS)^5^. Our previous data demonstrated that upregulation of helper type 2 (Th2), but not Th1, is important in IgG4-DS^6^,^7^. In addition, we previously identified that Th2-derived cytokines such as interleukin (IL)-4, IL-10, and IL-21 contribute to the pathogenesis of IgG4-DS, specifically facilitating the production of IgG4 and formation of ectopic germinal centers^7^,^8^. It should be noted that the abovementioned studies were conducted on salivary glands (SGs) from patients with IgG4-DS; however, the SG samples from these patients have high frequencies of typical IgG4-RD characteristics and are indicated as an ideal pathological manifestation of IgG4-RD^9^,^10^. Together, these studies have revealed that the main pathogenesis of IgG4-RD is related to Th2 cell and cytokine activation^11^,^12^.

To our knowledge, no published reports have investigated the mechanism of Th2 cell activation in IgG4-RD. Recently, IL-33, a member of the IL-1 cytokine family, has been shown to accelerate Th2 cell expression of the IL-33 receptor, ST2^13^. *In vitro*, IL-33 directly stimulates Th2 cells, mast cells, eosinophils, and basophils, resulting in Th2 cytokine production^14^. Several studies have reported that IL-33 has important biological functions in several immune-mediated diseases such as asthma, allergic rhinitis, cardiovascular disease, multiple sclerosis, rheumatoid arthritis, and SS^15^,^16^,^17^,^18^,^19^. Moreover, IL-33 is produced by epithelial cells, macrophages, dendritic cells (DCs), and mast cells^13^. In humans, at least two types of macrophages and DCs have been identified based on their function and reaction. The macrophages include the classically activated macrophage (M1), which is activated via CD68^+^ CD163^−^, and the alternatively activated macrophage (M2), which is activated via CD68^+^ CD163^+^^20^. The DCs include myeloid DC (mDC), which is activated by CD11c^high^ CD123^low^, and plasmacytoid DC (pDC), which is activated by CD11c^−^ CD123^high ^21.

Recent reports have indicated that the M1 macrophage is capable of stimulating the Th1 response via IL-6 and IFN-γ. Alternatively, the M2 macrophage is stimulated by the Th2 response, and promotes the Th2 reaction through IL-13 production^22^,^23^. Further, mDCs likely exert inhibitory effects on the development of Th1 inflammatory responses, while pDCs inhibit Th2 inflammatory responses^24^. However, the relationship between macrophages, DCs, and IgG4-RD has not yet been elucidated. Therefore, to clarify the contribution of IL-33 to the pathogenesis of IgG4-RD, we examined infiltrating cells expressing IL-33 in SGs from patients with IgG4-RD.

## Materials and Methods

### Ethics Statement

The study design and methods were approved by the Institutional Review Board of Center for Clinical and Translational Research of Kyushu University Hospital (IRB serial number: 25–287). The methods were carried out in accordance with the approved guidelines. All patients or their relatives gave their informed consent within written treatment contract on admission and therefore prior to their inclusion in the study.

### Study Participants

SG samples were collected from patients referred to the Department of Oral and Maxillofacial Surgery, Kyushu University Hospital between 2010 and 2014. Seven patients with IgG4-RD (five men and two women; mean age ± standard deviation (SD), 61.7 ± 12.1 years), 10 with primary SS (five men and five women; 55.6 ± 20.5 years), and 10 with oral squamous cell carcinoma (OSCC) as a control group (five men and five women; 58.4 ± 16.3 years) participated in this study. Clinical and serological profiles of patients with IgG4-RD are listed in Table 1.

Patients with IgG4-RD and SS underwent open SG biopsies, as described by Moriyama *et al*.^20^, while patients with OSCC underwent a neck dissection. SGs from patients with OSCC were histologically normal and exhibited no clinical evidence of metastasis. IgG4-RD were diagnosed according to both the “Comprehensive diagnostic criteria for IgG4-related disease”^2^ and “Diagnostic criteria for IgG4-DS”^4^. All patients with IgG4-RD showed characteristic histopathological findings including marked infiltration of IgG4-positive plasma cells, severe fibrosis, and formation of multiple ectopic GCs, and had never been treated with steroids or any other immunosuppressants. SS was diagnosed according to both the Research Committee on SS of the Ministry of Health and Welfare of the Japanese Government (1999)^25^ and the American-European Consensus Group criteria for SS^26^. All patients with SS had lymphocytic infiltration in the LSGs, had no other autoimmune diseases, and had never been treated with steroids or any other immunosuppressants. There was no documented history of HIV, HTLV-1, hepatitis B virus or hepatitis C virus infection in any of the patients. None of the patients had evidence of malignant lymphoma at the time of the study

### Immunohistochemical analysis

For immunohistochemical analysis, 4 μm formalin-fixed, paraffin-embedded SG sections were prepared and stained by a conventional avidin–biotin complex technique, as previously described^7^. Anti-ST2, -CD11c, -IL-4, and -IL-13 rabbit polyclonal antibodies were used to analyze ST2 (clone: HPA007406, Atlas, Stockholm), CD11c (clone: ab52632; Abcam, USA), IL-4 (clone: ab9622; Abcam, USA), and IL-13 (clone: HPA042421, Atlas, Stockholm) cytokines, respectively. Anti-IL-33, -CD68, -CD163, and -CD123 mouse monoclonal antibodies were used to analyze IL-33 (clone: Nessy-1, Enzo, Japan), CD123 (clone: NCL-CD123, Leica Biosystems, Germany), CD68 (clone: ab955; Abcam, USA), and CD163 (clone: NCL-CD163, Leica Biosystems, Germany) molecules, respectively. Sections were stained and evaluated according to previously described methods^27^.

### Double immunofluorescence of IL-33 and cell markers in SGs from patients with IgG4-RD

For double immunofluorescence analysis, 4 μm formalin-fixed and paraffin-embedded SGs from patients with IgG4-RD were prepared and stained. Sections were incubated with the primary antibody, IL-33 (Enzo), at room temperature for 2 h after blocking with 1% BSA blocking buffer for 1 h. Sections were then incubated with the secondary antibody, Alexa 488^®^ (1:100 dilution, Abcam, USA), for 30 min and washed well while avoiding light. Sections were blocked in 1% BSA blocking buffer for 40 min and then incubated with the primary antibodies against CD68 (Abcam), CD163 (clone: EDHu-1, AbD Serotec, USA), CD11c (Abcam), or CD123 (Leica) at room temperature for 2 h. After incubation, sections were incubated in Alexa 568^®^ secondary antibody (1:100 dilution, Abcam, USA) for 30 min at room temperature, washed well while avoiding light, mounted in VECTASHIELD^®^ with DAPI for nuclei staining (Vector Laboratories, USA), and stored in the dark. Images were taken using a Keyence microscope (BZ-9000 series) with the background fluorescence level set using negative controls as no detected nuclei.

### RNA extraction and complementary DNA (cDNA) synthesis

Total RNA was prepared from SGs by the acidified guanidinium–phenol–chloroform method as previously described^7^,^28^. One microgram of the total RNA preparation was then used for the synthesis of cDNA. Briefly, RNA was incubated for 1 h at 42 °C with 20 U of RNasinribonuclease inhibitor (Promega, Madison, WI, USA), 0.5 μg of oligo-1218 (Pharmacia, Uppsala, Sweden), 0.5 mM of each deoxyribonucleotide triphosphate (dNTP) (Pharmacia), 10 mM of dithiothreitol (DTT), and 100 U of RNase Reverse Transcriptase (Life Technologies, Gaithersburg, MD, USA).

### Quantitative estimation of mRNA by real-time PCR

mRNA levels of the cytokines and chemokines in SGs were quantified by real-time PCR, using Light Cycler Fast Start DNA Master SYBR Green III (Roche Diagnostics, Mannheim, Germany) in a Light Cycler real-time PCR instrument (version 3.5; Roche Diagnostics). In this study, the cytokines and cluster of differentiations were IL-33, ST2, IL-4, IL-13, CD68, CD163, CD11c, and CD123. Target mRNA levels were expressed relative to β-actin as the housekeeping gene. The primer sequences used were as follows: β-actin, forward 5′-GCA AAG ACC TGT ACG CCA AC-3′, reverse 5′-CTA GAA GCA TTT GCG GTG GA-3′, 260 bp; IL-33, forward 5′-ATG AGT CTC AAC ACC CCT CAA-3′, reverse 5′-CTG GTC TGG CAG TGG TTT TT-3′, 155 bp; ST2 forward 5′-ATT TGC ATG GCT TGA GAA GG-3′, reverse 5′-AGA GAA GCT CCC AGC AAA CA-3′, 240 bp; IL-4 forward 5′-AGC TGA TCC GAT TCC TGA AAC-3′, reverse 5′-TAC TCT GGT TGG CTT CCT TCA C-3′, 90 bp; IL-13 forward 5′-GGT CAA CAT CAC CCA GAA CC-3′, reverse 5′-TTT ACA AAC TGG GCC ACC TC-3′, 240 bp; CD68 forward 5′-TCA GAA TGC ATC CCT TCG AG-3′, reverse 5′-GAT GAG AGG CAG CAA GAT GG-3′, 199 bp; CD163 forward 5′-TGA TT CGG ACT TCT CTC TGG-3′, reverse 5′-ACT GGG CAG AGT GAA AGA TG-3′, 168 bp; CD11c forward 5′-TAC CTC ACC GGA CTC TGC TT-3′, reverse 5′-GGA GAA CTG CAT CAG GGA AA-3′, 228 bp; CD123 forward 5′-CCC AAC ATG ACT GCA AAG TG-3′, reverse 5′-CTA GAA GCA TTT GCG GTG GA-3′, 200 bp. All analyses were performed in triplicate.

### Cytokine determination by enzyme-linked immunosorbent assay (ELISA)

IL4, IL-13, and IL-33 were measured by ELISA (IL-4: ab181426; Abcam, IL-13: ab178014; Abcam, and IL-33: GWB-SKR038; Gen Way Biotech, USA), according to the manufacturer’s protocol. These results were compared with 12 non-smoking volunteers (healthy controls) who had no sicca or clinical or laboratory evidence of systemic disease. All analyses were performed in triplicate.

### Statistics

Statistical significance of the differences between groups was determined using the following methods. A Mann-Whitney *U* test was used when populations were not normally distributed in the detection of mRNA expression levels^7^,^28^. A non-parametric Spearman test was used for the correlation analysis. All statistical analyses were performed with JMP software, version 8 (SAS Institute, Cary, NC, USA). A *P* value less than 0.05 was considered statistically significant.

## Results

### Expression of IL-33, ST2, and Th2 cytokines in SGs

**Figure 1:**
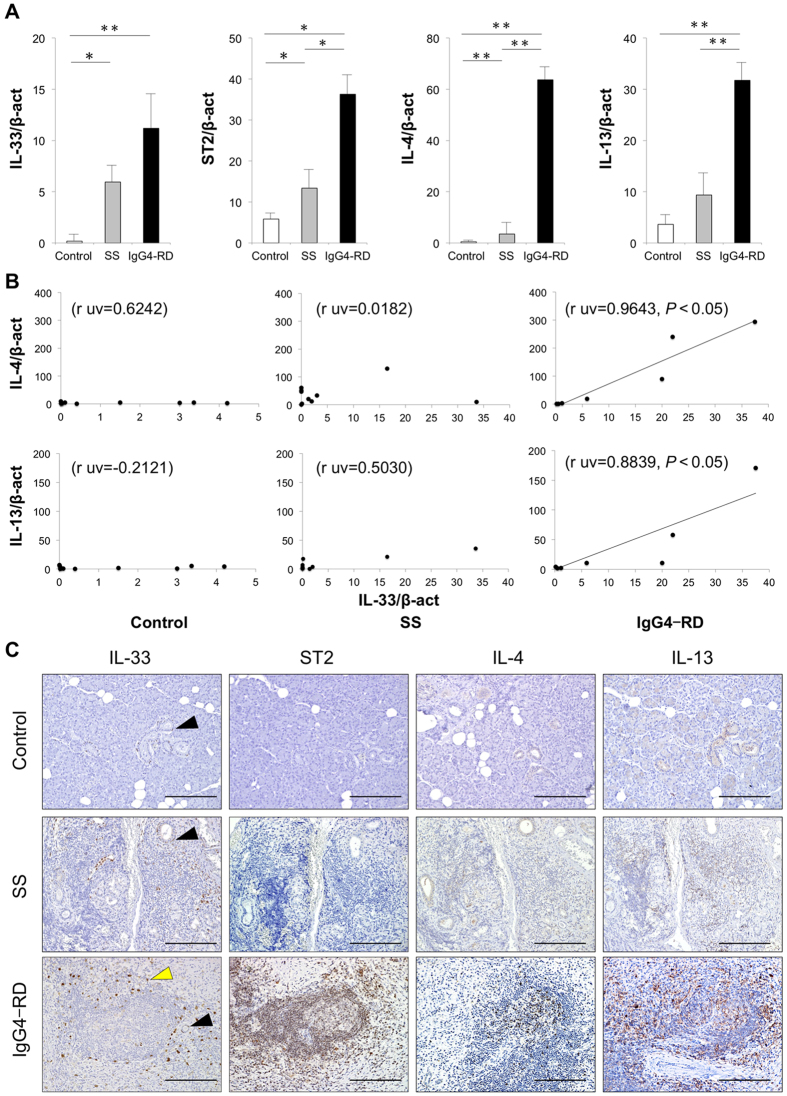
The expression of IL-33 and related molecules was higher in patients with IgG4-related disease (IgG4-RD). (**A**) mRNA expression levels of IL-33 and related molecules was examined in salivary glands (SGs) from controls (n = 10), and patients with Sjögren’s syndrome (SS) (n = 10) and IgG4-RD (n = 7). Molecules were quantitatively estimated as described in the Methods section, and significant differences between groups were determined by the Mann–Whitney *U* test (*p < 0.05, **p < 0.01). (**B**) Correlation between mRNA expression levels of IL-33 and related molecules in SGs from controls, as well as patients with SS and IgG4-RD. Statistical significance of differences between groups was determined by Spearman’s rank correlation (*p* < 0.05). (**C**) Distribution of IL-33 and related molecules in SGs from representative controls and patients with SS and IgG4-RD. Counterstaining was performed with Mayer’s hematoxylin (blue). IL-33 positive ductal epithelial cells are indicated by block arrows, while IL-33 positive cells are indicated by yellow arrows. Scale bars, 100 μm.

The mRNA expression levels of IL-33, ST2, and IL-4 in SGs from patients with SS and IgG4-RD were significantly higher than those in controls. Furthermore, the mRNA levels of ST2, IL-4, and IL-13 in patients with IgG4-RD were significantly higher than those from patients with SS (Fig. 1A). The relationships between mRNA expression levels of IL-33 and Th2 cytokines (IL-4, IL-13) in SGs were examined, and the mRNA expression of IL-33 was positively correlated with Th2 cytokines in SGs from patients with IgG4-RD but not with Th2 cytokines in SGs from patients with SS and controls (Fig. 1B). To evaluate the distribution of IL-33, ST2, and Th2 cytokines, representative histological findings in the SG specimens from patients with SS and IgG4-RD and from controls are shown in Fig. 1C. Expression of IL-33 was detected in all samples in the ductal epithelial cells, identified morphologically and indicated by black arrows. Interestingly, IL-33 was also detected in infiltrating lymphocytes around ectopic germinal centers (GCs) in patients with IgG4-RD, as indicated by yellow arrows in Fig. 1C. Expression of ST2, IL-4, and IL-13 was detected in infiltrating lymphocytes around ectopic GCs from patients with SS and IgG4-RD, but not in controls. In addition, patients with IgG4-RD showed strong infiltration of these positive cells in comparison to patients with SS.

### Expression of IL-33-producing cells in SGs

**Figure 2:**
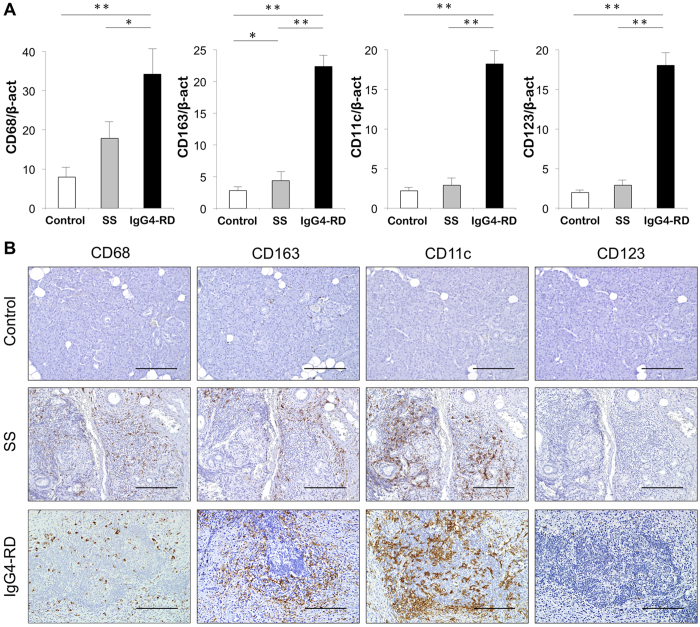
Expression and localization of innate immune cells in SGs. (**A**) mRNA expression levels of innate immune cells in SGs from controls (n = 10), and patients with SS (n = 10) and IgG4-RD (n = 7). Expression levels of innate immune cells markers for macrophages (CD68), M2 macrophages (CD163), mDC (CD11c), and pDC (CD123) were estimated quantitatively as described in the Methods section. Statistically significant differences between groups were determined by Mann–Whitney *U* tests (*p < 0.05, **p < 0.01). (**B**) Distribution of innate immune cells in SGs from representative controls and patients with SS and IgG4-RD. Counterstaining was performed with Mayer’s hematoxylin (blue). Scale bars, 100 μm.

As IL-33 is considered to be produced by epithelial cells and antigen-presenting cells (APCs), such as macrophages and DCs^13^, we examined the expression of IL-33-producing cells in SGs from patients with SS and IgG4-RD, as well as in controls. The mRNA levels of CD68, CD163, CD11c, and CD123 were significantly higher in patients with IgG4-RD than in the two other evaluated groups (Fig. 2A). Specimens were also immunohistochemically examined to clarify the distribution of IL-33-producing cells, including macrophagess (CD68, CD163), and DCs (CD11c, CD123), in the SGs from patients with SS and IgG4-RD, as well as controls. Expression of CD68 and CD163 was strongly detected around ectopic GCs in SGs from patients with IgG4-RD, whereas CD68 and CD163 were detected around ductal epithelial cells in patients with SS. Expression of CD11c was strongly detected in/around ectopic GCs in patients with IgG4-RD, whereas CD11c was detected around ductal epithelial cells in patients with SS. Finally, expression of CD123 was slightly detected in/around ectopic GCs in patients with IgG4-RD, but CD123 was rarely seen in patients with SS. All of these markers were rarely seen in controls (Fig. 2B).

### Co-localization of IL-33 and cell markers in SGs from patients with IgG4-RD

**Figure 3:**
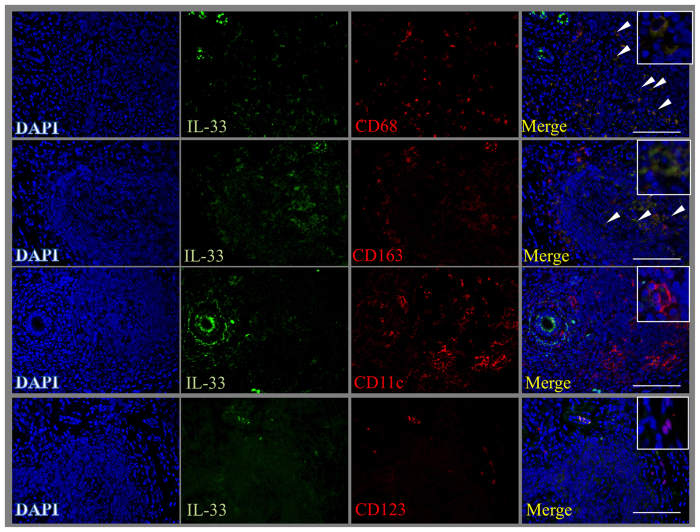
M2 macrophages produce IL-33 in SGs from patients with IgG4-RD. Double immunofluorescence staining performed with IL-33 (green), innate immune cells markers for macrophages (CD68), M2 macrophages (CD163), mDC (CD11c), and pDC (CD123) (red), and DAPI for nuclei staining (blue), as described in the Methods section. Scale bars, 50 μm.

To clarify which cells produced IL-33, double immunofluorescence staining with IL-33 (green) and CD68, CD163, CD11c, or CD123 (red) was performed. Using this approach, we observed that IL-33-positive cells co-localized with CD68- and CD163-positive cells, indicated by white arrows, and only partly co-localized with CD11c- or CD123-positive cells (Fig. 3). Therefore, macrophages, especially M2 macrophages, might be responsible for the production of IL-33 in SGs from patients with IgG4-RD.

### Serum concentration of IL-33 and Th2 cytokines

**Figure 4:**
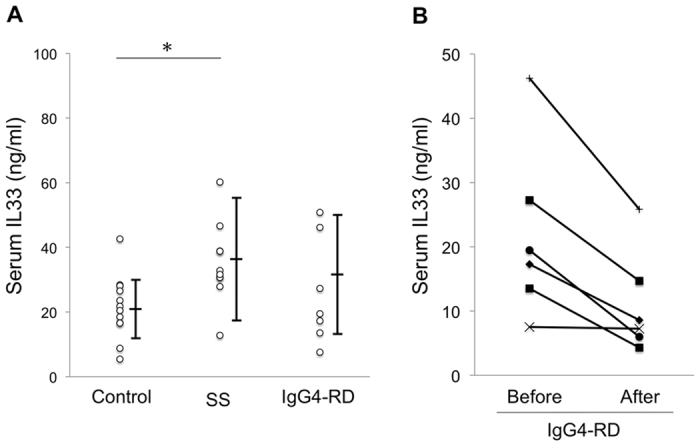
Serum concentration of IL-33. (**A**) Serum levels of IL-33 were measured in healthy controls (n = 12), and patients with SS (n = 10) and IgG4-RD (n = 7). (**B**) Serum levels of IL-33 were measured in 6 patients with IgG4-RD who were treated by corticosteroid after final diagnosis. Significant differences between groups were determined by the Mann–Whitney *U* test (*p < 0.05).

Recent study reported that serum IL-33 and ST2 were significantly elevated in pSS patients in comparison with healthy controls^18^. Therefore, we measured the serum concentration of IL-33 and Th2 cytokines (IL-4 and IL-13) in patients with SS, IgG4-RD, and healthy controls by ELISA. Serum IL-33 levels in patients with pSS were higher than that in controls (Fig. 4A), and IgG4-RD showed the decrease in serum IL33 after steroid therapy (Fig. 4B). On the other hand, there was no significant difference in serum IL-4 and IL-13 levels between IgG4-RD patients and controls (Supplementary Figure 1).

## Discussion

IgG4-RD is well known as a systemic disorder that is characterized by elevated serum IgG4, marked infiltration of IgG4-positive plasma cells, and severe fibrosis with hyperplastic eGCs in affected lesions. Although many basic studies on IgG4-RD have been reported in the past 10 years, the pathogenic mechanism of the disease remains unclear. Recent studies have reported that IgG4-RD is a Th2-dominant disease and that aberrant activation of the Th2 immune response plays a key role in its pathogenesis^6^,^7^,^8^,^29^,^30^. In this study, we focused on IL-33 as a recently identified cytokine that enhances Th2 cytokine production.

Recent studies indicated the involvement of the IL-33/ST2 axis in the pathogenesis of pSS^18^,^19^. They found that serum IL-33 and ST2 levels were increased in pSS patients and that IL-33 producing cells were mainly epithelial cells in SGs from patients with pSS. Our current data were consistent with these previous reports, and reveal that expression of IL-33 in SGs from patients with IgG4-RD was detected not only in ductal epithelial cells, but also around ectopic GCs. It is found that IL-33 was mainly expressed in epithelial cells and produced by a wide variety of cell types including DCs, macrophages, fibroblasts, mast cells, and osteoblasts^31^. Here, to confirm the IL-33-producing cells around ectopic GCs, we analyzed the expression of macrophages and DCs and found that IL-33 could be localized to M2 macrophages. In addition, mRNA expression of IL-33 was positively correlated with that of Th2 cytokines in patients with IgG4-RD. These results indicate that the majority of IL-33-producing cells in IgG4-RD might be M2 macrophages, which are deeply involved in the activation of Th2 immune responses.

With regard to DCs, recent studies that pDCs in SGs ware associated with the pathological processes of acute/chronic salivary inflammation in SS and IgG4-RD^32^,^33^. In our current study, we found that IL-33-positive cells partly co-localized with CD11c- or CD123-positive DCs in SGs from patients with IgG4-RD, but IL-33 and DCs were rarely seen in SS. Maria NI, *et al*.^34^ divided pSS patients according to the mRNA expression of IFN type I in PBMC, and demonstrated that pDCs were detected in SGs from only IFN-positive pSS. Considering to these reports, our current data (Fig. 2B) might be reflect to the results of IFN-negative pSS.

We previously examined M1 and M2 macrophage subsets in SGs from patients with SS, chronic sialoadenitis, and IgG4-RD, and indicated that the amount and frequency of M2 macrophages in patients with IgG4-RD were significantly higher than those in controls, as well as in patients with chronic sialoadenitis and SS. These findings were mirrored by other lesions in the LG, pancreas, pleura, and prostate gland from patients with IgG4-RD^27^. Furthermore, Fukui *et al*.^35^ detected M2 macrophages expressing toll-like receptor 7 (TLR7) in patients with IgG4-related autoimmune pancreatitis. Alveolar macrophages are the primary source of increased IL-33 levels during infection, and an *in vitro* stimulation of these cells with a TLR7 agonist induces IL-33 expression^36^. In the current study, we also detected a number of SGs expressing TLR7 from patients with IgG4-RD. Further, these SGs were shown to co-localize with CD163-positive cells (Supplemental Fig. 2), indicating that M2 macrophages activated by the TLR7 pathway might play a crucial role in IgG4-RD as well as IgG4-related AIP.

In our previous study, gene expression in SGs from patients with IgG4-RD, chronic sialoadenitis and controls was analyzed via DNA microarrays. Using this approach, the macrophage receptor with collagenous structure (MARCO), one of the scavenger receptors^37^, was identified as a disease-associated molecule in patients with IgG4-RD. Moreover, immunohistochemical analysis has confirmed that the expression patterns of MARCO are similar to those of M2 macrophage marker CD163^10^. MARCO, a scavenger receptor expressed by macrophages^38^, has been reported to play an important role in the innate immune response by mediating ligand binding and phagocytosis. Further, this receptor can recognize various ligands including bacterial lipopolysaccharide (LPS), apoptotic cells, lung pathogens, environmental particles, and nanomaterials^39^.

Taken together with the previous reports, the current results indicate that M2 macrophages may contribute to the initiation or maintenance of IgG4-RD via IL-33 production. We thus made the following hypothesis regarding the pathogenic process in IgG4-RD: M2 macrophages recognize certain exogenous or endogenous molecules through binding to pattern-recognition receptors, including TLR7 and MARCO. Following their binding to these receptors, M2 macrophages promote the production of IL-33, which precipitates an exaggerated Th2 immune responses and the pathology noted in IgG4-RD (Supplementary Figure 3).

On the other hand, other Th subsets (regulatory T cells, T follicular helper (Tfh) cells, and CD4+ cytotoxic T lymphocytes) and innate immune cells (DCs and basophils) have recently received increasing attention with regard to the pathogenesis of IgG4-RD^29^,^40^,^41^. Maehara T, *et al*.^42^ reported that the ratio of CD4+ GZMA+ CTLs in SMGs from patients with IgG4-DS correlated with serum IgG4 concentrations and the number of affected organs. Therefore, IgG4-RD might be initiated and progressed by the interconnected network of various Th subsets. A literature Summary on the etiology and pathogenesis of IgG4-DS and SS is shown in Table 2.

In conclusion, we confirmed IL-33 overexpression in M2 macrophages clustered around ectopic GCs in SGs from patients with IgG4-RD. However, functional assays are needed to elucidate the mechanism of M2 macrophage-mediated IL-33 production. Although glucocorticoids are effective and the standard treatment for IgG4-RD, Yamamoto *et al*.^43^ reported that almost half of patients with IgG4-RD experience disease relapse during long-term steroid therapy. A more thorough understanding of the role of M2 macrophages in IgG4-RD could lead to the establishment of a mouse model of IgG4-RD and to the eventual development of novel pharmacological strategies to interrupt IL-33/ST2 signaling as a further means of inhibiting disease initiation or progression.

## Additional Information

**How to cite this article**: Furukawa, S. *et al*. Interleukin-33 produced by M2 macrophages and other immune cells contributes to Th2 immune reaction of IgG4-related disease. *Sci. Rep.* **7**, 42413; doi: 10.1038/srep42413 (2017).

**Publisher's note:** Springer Nature remains neutral with regard to jurisdictional claims in published maps and institutional affiliations.

## Supplementary Material

Supplementary Figures

## Figures and Tables

**Table 1:** Clinical and serological findings of seven patients with IgG4-related disease (IgG4-RD).

No.	Age	Sex	Disease Duration	Complications							Histological findings				Gum test	Saxon test	Serological tests								
					Swollen glands						IgG4/IgG	IgG4+ cells	Complaint				RF	ANA	IgG	IgG4	IgA	IgE	IgM	Anti-SSA	Anti-SSB
					LG	PG	SMG	SLG	PLG	LSG	(%)	(/HPF)	Dry eye	Dry mouth	(ml/10 min)	(g/2 min)	(U/ml)		(mg/dl)	(mg/dl)	(mg/dl)	(U/ml)	(mg/dl)	(U/ml)	(U/ml)
1	69	M	3 M	HT	+	−	+	+	−	+	61.2	32	−	−	6.0	1.8	ND	±	***1663***	***458***	97	60	79	−	−
2	68	F	9 M	AIP	−	−	+	+	−	+	52.3	42	−	−	6.0	2.2	5	***160***	***6758***	***1500***	78	13	81	−	−
3	39	M	2 Y	pollinosis	+	−	+	−	−	+	64.2	77	−	−	16.4	4.7	5	−	1534	***188***	170	***1619***	99	−	−
4	69	M	4 M	AIP	−	−	+	+	−	+	63.2	79	−	−	12.0	6.8	ND	ND	***1675***	***484***	229	***283***	44	−	−
5	74	M	4 M	AIP	+	−	+	+	−	+	50.0	85	−	−	10.2	3.8	ND	40	***4217***	***524***	177	29	60	−	−
6	58	F	6 M	−	−	−	+	+	−	−	73	28	−	−	12.0	3.6	4	***80***	1188	***151***	193	***178***	56	−	−
7	55	M	3 Y	AIP, rectal cancer	+	−	+	+	−	−	70	18	−	−	7.4	3.2	4	−	***2092***	***510***	148	ND	70	−	−

Abbreviations: LG, lachrymal gland; PG, parotid gland; SMG, submandibular gland; SLG, sublingual gland; PLG, palatine gland; LSG, labial salivary gland; HPF, high power field; RF, rheumatoid factor; ANA, antinuclear antibody; HT, hyper tension; AIP, autoimmune pancreatitis; +, positive; −, negative; ND, not done; bold italics indicate higher than normal values.

**Table 2:** A comprehensive list of references on IgG4-related dacryoadenitis and sialoadenitis (IgG4-DS) and Sjogren’s syndrome (SS).

Principal findings	Reference
IgG4-DS (IgG4-RD)	
● Peripheral CD4. T cells from patients with IgG4-DS reveal the deviation of the Th1/Th2 (IFN-γ/IL-4) balance to Th2.	6
● Th2 and regulatory immune reactions play a key role of IgG4 production in IgG4-RD.	7, 29
● Overexpression of IL-21 promotes germinal center formation and IgG4 production in SGs.	8
● CD4+ cytotoxic T lymphocytes in SGs from patients with IgG4-RD positively correlate with serum IgG4 concentrations and the number of affected organ.	42
● IL-10 and CCL18 secreted by preferential M2 macrophages play a key role in the development of severe fibrosis in IgG4-DS.	27
● M2 macrophages might contribute to the initiation of IgG4-RD via MARCO identified as a disease-associated molecule by DNA microarray.	10
● Oligoclonal expansions of IgG4-expressing plasmablasts are involved in active and relapsing IgG4-RD.	44
● Peripheral Tfh2 cells from patients with IgG4-RD correlate with number of plasmablast and elevated serum levels of IgG4.	40
SS	
● SS is initiated and/or maintained by Th1 and Th17 cells, progress in association with Th2 cells.	
● IL-21 secreted by Tfh cells promotes germinal centers formation in SGs of patients with pSS.	28, 45
● pDCs in SGs are associated with the pathological processes of acute/chronic salivary inflammation in SS via type I IFN-CXCL13 axis.	32, 33
● IL-33 expression in epithelial cells of SGs is upregulated, acting in concerted fashion with IL-12 and IL-23 to trigger the secretion of IFN-γ by NK and NKT cells.	18
● Serum levels of IL-33 and ST2 were significantly elevated in pSS patients, especially in patients with ILD.	19

Abbreviations: Th, T helper; SG, salivary gland; MARCO, macrophage receptor with collagenous structure; Tfh, follicular helper T; pDC, plasmacytoid dendritic cell; NK, natural killer; pSS, primary SS; ILD, interstitial lung disease.

## References

[b1] Stone J. H. . IgG4-Related Disease. Int J Rheumatol 2013, 532612 (2013). 23401693 10.1155/2013/532612 PMC3562585

[b2] Umehara H. . Comprehensive diagnostic criteria for IgG4-related disease (IgG4-RD), 2011. Mod Rheumatol 22(1), 21–30 (2012). 22218969 10.1007/s10165-011-0571-z

[b3] Morgan W. S. & Castleman B. A clinicopathologic study of Mikulicz’s disease. Am J Pathol 29(3), 471–503 (1953). 13040489 PMC1937437

[b4] Yamamoto M., Takahashi H., Sugai S. & Imai K. Clinical and pathological characteristics of Mikulicz’s disease (IgG4-related plasmacytic exocrinopathy). Autoimmun Rev 4(4), 195–200.(2005). 15893711 10.1016/j.autrev.2004.10.005

[b5] Stone J. H. . Recommendations for the nomenclature of IgG4-related disease and its individual organ system manifestations. Arthritis Rheum 64(10), 3061–3067 (2012). 22736240 10.1002/art.34593 PMC5963880

[b6] Miyake K. . Peripheral CD4+ T cells showing a Th2 phenotype in a patient with Mikulicz’s disease associated with lymphadenopathy and pleural effusion. Mod Rheumatol 18(1), 86–90 (2008). 18094933 10.1007/s10165-007-0010-3

[b7] Tanaka A. . Th2 and regulatory immune reactions contribute to IgG4 production and the initiation of Mikulicz disease. Arthritis Rheum 64(1), 254–263 (2012). 21898360 10.1002/art.33320

[b8] Maehara T. . Interleukin-21 contributes to germinal centre formation and immunoglobulin G4 production in IgG4-related dacryoadenitis and sialoadenitis, so-called Mikulicz’s disease. Ann Rheum Dis 71(12), 2011–2019 (2012). 22753386 10.1136/annrheumdis-2012-201477

[b9] Moriyama M. . The diagnostic utility of biopsies from the submandibular and labial salivary glands in IgG4-related dacryoadenitis and sialoadenitis, so-called Mikulicz’s disease. Int J Oral Maxillofac Surg 43(10), 1276–1281 (2014). 25062551 10.1016/j.ijom.2014.06.014

[b10] Ohta M. . DNA Microarray Analysis of Submandibular Glands in IgG4-Related Disease Indicates a Role for MARCO and Other Innate Immune-Related Proteins. Medicine (Baltimore) 95(7), e2853 (2016). 26886650 10.1097/MD.0000000000002853 PMC4998650

[b11] Tsuboi H. . DNA microarray analysis of labial salivary glands in IgG4-related disease: comparison with Sjogren’s syndrome. Arthritis Rheumatol 66(10), 2892–2899 (2014). 24943710 10.1002/art.38748

[b12] Takeuchi M. . T helper 2 and regulatory T-cell cytokine production by mast cells: a key factor in the pathogenesis of IgG4-related disease. Mod Pathol 27(8), 1126–1136 (2014). 24390219 10.1038/modpathol.2013.236

[b13] Schmitz J. . IL-33, an interleukin-1-like cytokine that signals via the IL-1 receptor-related protein ST2 and induces T helper type 2-associated cytokines. Immunity 23(5), 479–490 (2005). 16286016 10.1016/j.immuni.2005.09.015

[b14] Louten J. . Endogenous IL-33 enhances Th2 cytokine production and T-cell responses during allergic airway inflammation. Int Immunol 23(5), 307–315 (2011). 21422152 10.1093/intimm/dxr006

[b15] Eiwegger T. & Akdis C. A. IL-33 links tissue cells, dendritic cells and Th2 cell development in a mouse model of asthma. Eur J Immunol 41(6), 1535–1538 (2011). 21618506 10.1002/eji.201141668

[b16] Christophi G. P., ey al. Interleukin-33 upregulation in peripheral leukocytes and CNS of multiple sclerosis patients. Clin Immunol 142(3), 308–319 (2012). 22189043 10.1016/j.clim.2011.11.007 PMC3288946

[b17] Kamekura R. . The role of IL-33 and its receptor ST2 in human nasal epithelium with allergic rhinitis. Clin Exp Allergy 42(2), 218–228 (2012). 22233535 10.1111/j.1365-2222.2011.03867.x

[b18] Awada A. . Potential involvement of the IL-33-ST2 axis in the pathogenesis of primary Sjogren’s syndrome. Ann Rheum Dis 73(6), 1259–63 (2014). 24385203 10.1136/annrheumdis-2012-203187

[b19] Zhao L. . Potential contribution of interleukin-33 to the development of interstitial lung disease in patients with primary Sjogren’s Syndrome. Cytokine 64(1), 22–4 (2013). 23910012 10.1016/j.cyto.2013.07.006

[b20] Gordon S. Alternative activation of macrophages. Nat Rev Immunol 3(1), 23–35 (2003). 12511873 10.1038/nri978

[b21] Hashizume H., Horibe T., Yagi H., Seo N. & Takigawa M. Compartmental imbalance and aberrant immune function of blood CD123+ (plasmacytoid) and CD11c+ (myeloid) dendritic cells in atopic dermatitis. J Immunol 174(4), 2396–2403 (2005). 15699176 10.4049/jimmunol.174.4.2396

[b22] Gordon S. & Martinez F. O. Alternative activation of macrophages: mechanism and functions. Immunity 32(5), 593–604 (2010). 20510870 10.1016/j.immuni.2010.05.007

[b23] Martinez F. O. & Gordon S. The M1 and M2 paradigm of macrophage activation: time for reassessment. F1000Prime Rep 6, 13 (2014) 24669294 10.12703/P6-13 PMC3944738

[b24] Charbonnier A. S. . Der p 1-pulsed myeloid and plasmacytoid dendritic cells from house dust mite-sensitized allergic patients dysregulate the T cell response. J Leukoc Biol 73(1), 91–99 (2003). 12525566 10.1189/jlb.0602289

[b25] Fujibayashi T., Sugai S., Miyasaka N., Hayashi Y. & Tsubota K. Revised Japanese criteria for Sjogren’s syndrome. availability and validity. Mod Rheumatol 2004;14(6), 425–434. (1999). 10.3109/s10165-004-0338-x 24387718

[b26] Vitali C. . Classification criteria for Sjogren’s syndrome: a revised version of the European criteria proposed by the American-European Consensus Group. Ann Rheum Dis 61(6), 554–558 (2002). 12006334 10.1136/ard.61.6.554 PMC1754137

[b27] Furukawa S. . Preferential M2 macrophages contribute to fibrosis in IgG4-related dacryoadenitis and sialoadenitis, so-called Mikulicz’s disease. Clin Immunol 156(1), 9–18 (2014). 25450336 10.1016/j.clim.2014.10.008

[b28] Moriyama M. . Cytokine/chemokine profiles contribute to understanding the pathogenesis and diagnosis of primary Sjogren’s syndrome. Clin Exp Immunol 169(1), 17–26 (2012). 22670774 10.1111/j.1365-2249.2012.04587.x PMC3390469

[b29] Zen Y. . Th2 and regulatory immune reactions are increased in immunoglobin G4-related sclerosing pancreatitis and cholangitis. Hepatology 45(6), 1538–1546 (2007). 17518371 10.1002/hep.21697

[b30] Tsuboi H. . Analysis of IgG4 class switch-related molecules in IgG4-related disease. Arthritis Res Ther 14(4), R171 (2012). 22824292 10.1186/ar3924 PMC3580565

[b31] Mirchandani A. S., Salmond R. J. & Liew F. Y. Interleukin-33 and the function of innate lymphoid cells. Trends Immunol 33(8), 389–396 (2012). 22609147 10.1016/j.it.2012.04.005

[b32] Zhao J. . Association of plasmacytoid dendritic cells with B cell infiltration in minor salivary glands in patients with Sjogren’s syndrome. Mod Rheumatol 26(5), 716–24 (2016). 26706891 10.3109/14397595.2015.1129694

[b33] Arai Y. . Plasmacytoid Dendritic Cell Activation and IFN-alpha Production Are Prominent Features of Murine Autoimmune Pancreatitis and Human IgG4-Related Autoimmune Pancreatitis. J Immunol 195(7), 3033–44 (2015). 26297761 10.4049/jimmunol.1500971

[b34] Maria N. I. . Contrasting expression pattern of RNA-sensing receptors TLR7, RIG-I and MDA5 in interferon-positive and interferon-negative patients with primary Sjögren’s syndrome. *Ann Rheum Dis* 2016 Sep 26. [Epub ahead of print] 10.1136/annrheumdis-2016-209589 27672125

[b35] Fukui Y. . Possible involvement of Toll-like receptor 7 in the development of type 1 autoimmune pancreatitis. J Gastroenterol 50(4), 435–44 (2015). 25005350 10.1007/s00535-014-0977-4

[b36] Chang Y. J. . Innate lymphoid cells mediate influenza-induced airway hyper-reactivity independently of adaptive immunity. Nat Immunol 12(7), 631–638 (2011). 21623379 10.1038/ni.2045 PMC3417123

[b37] Murthy S. . Alternative activation of macrophages and pulmonary fibrosis are modulated by scavenger receptor, macrophage receptor with collagenous structure. FASEB J 29(8), 3527–3536 (2015). 25953850 10.1096/fj.15-271304 PMC4511206

[b38] Elomaa O. . Cloning of a novel bacteria-binding receptor structurally related to scavenger receptors and expressed in a subset of macrophages. Cell 80(4), 603–609 (1995). 7867067 10.1016/0092-8674(95)90514-6

[b39] Jing J. . Role of macrophage receptor with collagenous structure in innate immune tolerance. J Immunol 190(12), 6360–6367 (2013). 23667110 10.4049/jimmunol.1202942 PMC3679202

[b40] Akiyama M. . Number of Circulating Follicular Helper 2 T Cells Correlates With IgG4 and Interleukin-4 Levels and Plasmablast Numbers in IgG4-Related Disease. Arthritis Rheumatol 67(9), 2476–81 (2015). 25989153 10.1002/art.39209

[b41] Mattoo H. . Clonal expansion of CD4(+) cytotoxic T lymphocytes in patients with IgG4-related disease. J Allergy Clin Immunol 138(3), 825–38 (2016). 26971690 10.1016/j.jaci.2015.12.1330 PMC5014627

[b42] Maehara T. . Lesional CD4+ IFN-γ+ cytotoxic T lymphocytes in IgG4-related dacryoadenitis and sialoadenitis. Ann Rheum Dis 2016 Jun 29. [Epub ahead of print] 10.1136/annrheumdis-2016-209139 PMC5435236 27358392

[b43] Yamamoto M. . Evaluation and Clinical Validity of a New Questionnaire for Mikulicz’s Disease. Int J Rheumatol 2012, 283459 (2012). 22649453 10.1155/2012/283459 PMC3357487

[b44] Matto H. . De novo oligoclonal expansions of circulating plasmablasts in active and relapsing IgG4-related disease. J Allergy Clin Immunol 134(3), 679–87 (2014). 24815737 10.1016/j.jaci.2014.03.034 PMC4149918

[b45] Maehara T. . Selective localization of T helper subsets in labial salivary glands from primary Sjögren’s syndrome patients. Clin Exp Immunol 169(2), 89–99 (2012). 22774983 10.1111/j.1365-2249.2012.04606.x PMC3406368

