# Supplementary material for: Interleukin-33 produced by M2 macrophages and other immune cells contributes to Th2 immune reaction of IgG4-related disease
Source: Sci Rep. 2017 Feb 13;7:42413. doi: 10.1038/srep42413 (PMC5304322; doi:10.1038/srep42413)
Supplement: Supplementary Figures [file srep42413-s1.pdf]

Interleukin-33 produced by M2 macrophages and other immune cells contributes to immune reaction of IgG4-related disease

Sachiko Furukawa, DDS, PhD[1], Masafumi Moriyama, DDS, PhD[1,2], Kensuke Miyake, MD, PhD[3], Hitoshi Nakashima, MD, PhD[4], Akihiko Tanaka, DDS, PhD[1], Takashi Maehara, DDS, PhD[1], Mana Iizuka-Koga, PhD[5,6], Hiroto Tsuboi, MD, PhD[5], Jun-Nosuke Hayashida, DDS, PhD[1], Noriko Ishiguro, DDS[1], Masaki Yamauchi, DDS[1], Takayuki Sumida, MD, PhD[5], and Seiji Nakamura, DDS, PhD[1]

[1] Section of Oral and Maxillofacial Oncology, Division of Maxillofacial Diagnostic and Surgical Sciences, Faculty of Dental Science, Kyushu University, Fukuoka, Japan

[2] OBT Research Center, Faculty of Dental Science, Kyushu University, Japan

[3] Division of Innate Immunity, Department of Microbiology and Immunology, Institute of Medical Science, University of Tokyo, Tokyo, Japan

[4] Division of Nephrology and Rheumatology, Department of Internal Medicine, Faculty of Medicine, Fukuoka University, Fukuoka, Japan

[5] Department of Internal Medicine, Faculty of Medicine, University of Tsukuba, Tsukuba, Japan

[6] Department of Microbiology and Immunology, Keio University School of Medicine, Tokyo, Japan

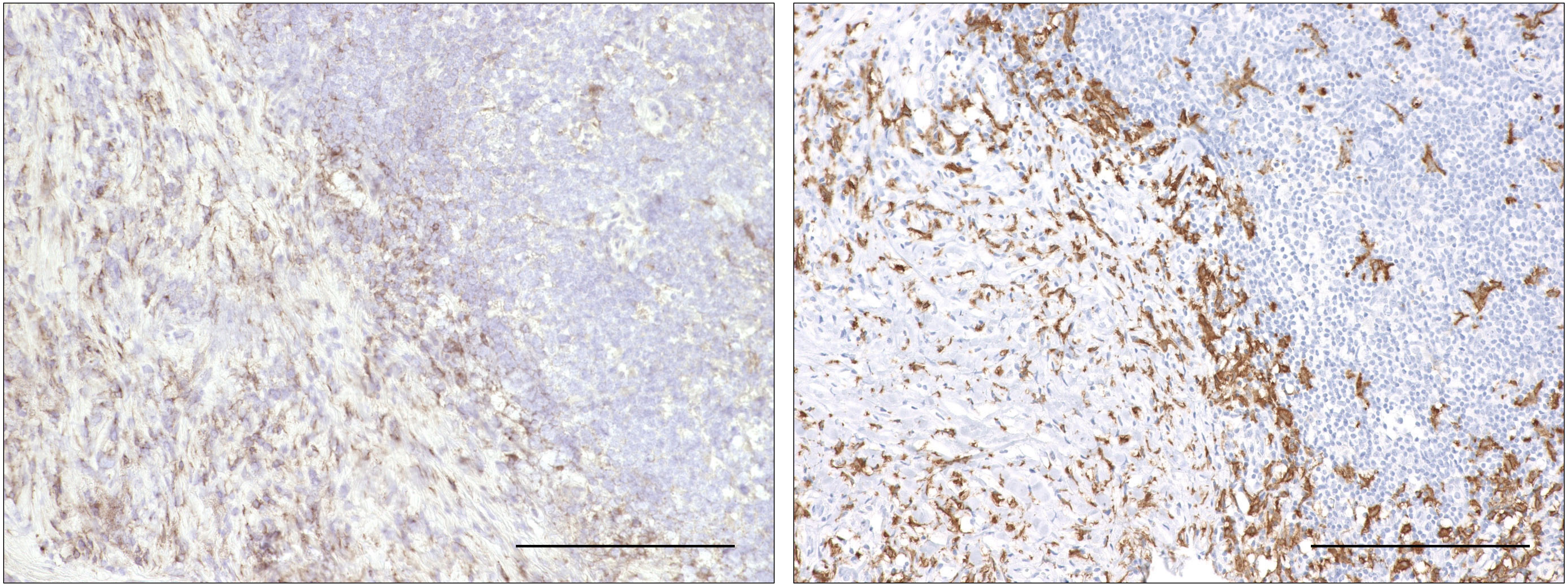

Supplementary Figure 1. Expression and localization of TLR7 and CD163 in SG from a representative patient with IgG4-RD. Counterstaining was performed with Mayer's hematoxylin (blue). Scale bars, 50 μm.

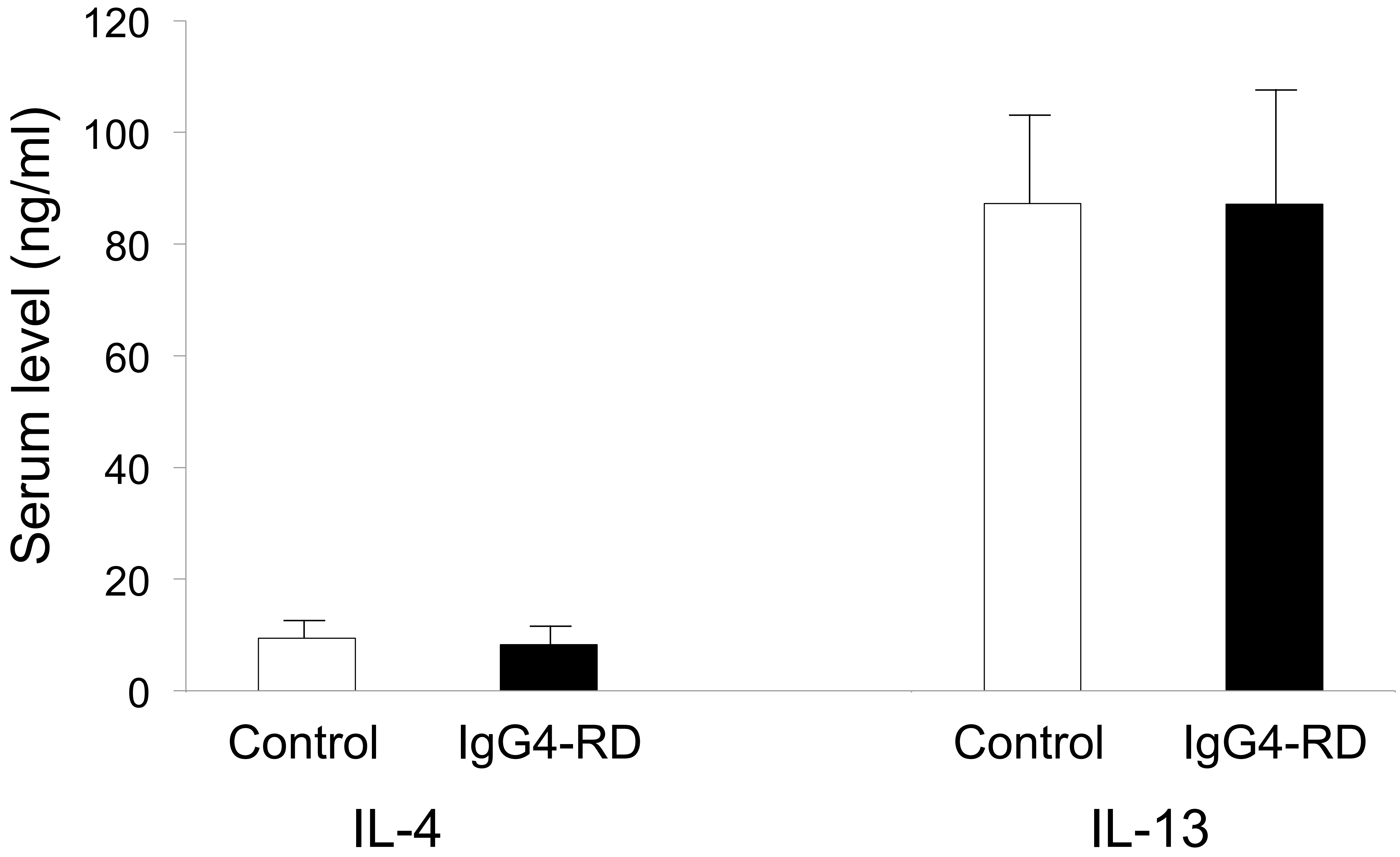

Supplementary Figure 2. Serum concentration of Th2 cytokines. Serum levels of IL-4 and IL-13 were measured in healthy controls (n = 12) and patients with IgG4-RD (n = 7). Significant differences between groups were determined by the Mann–Whitney *U* test.

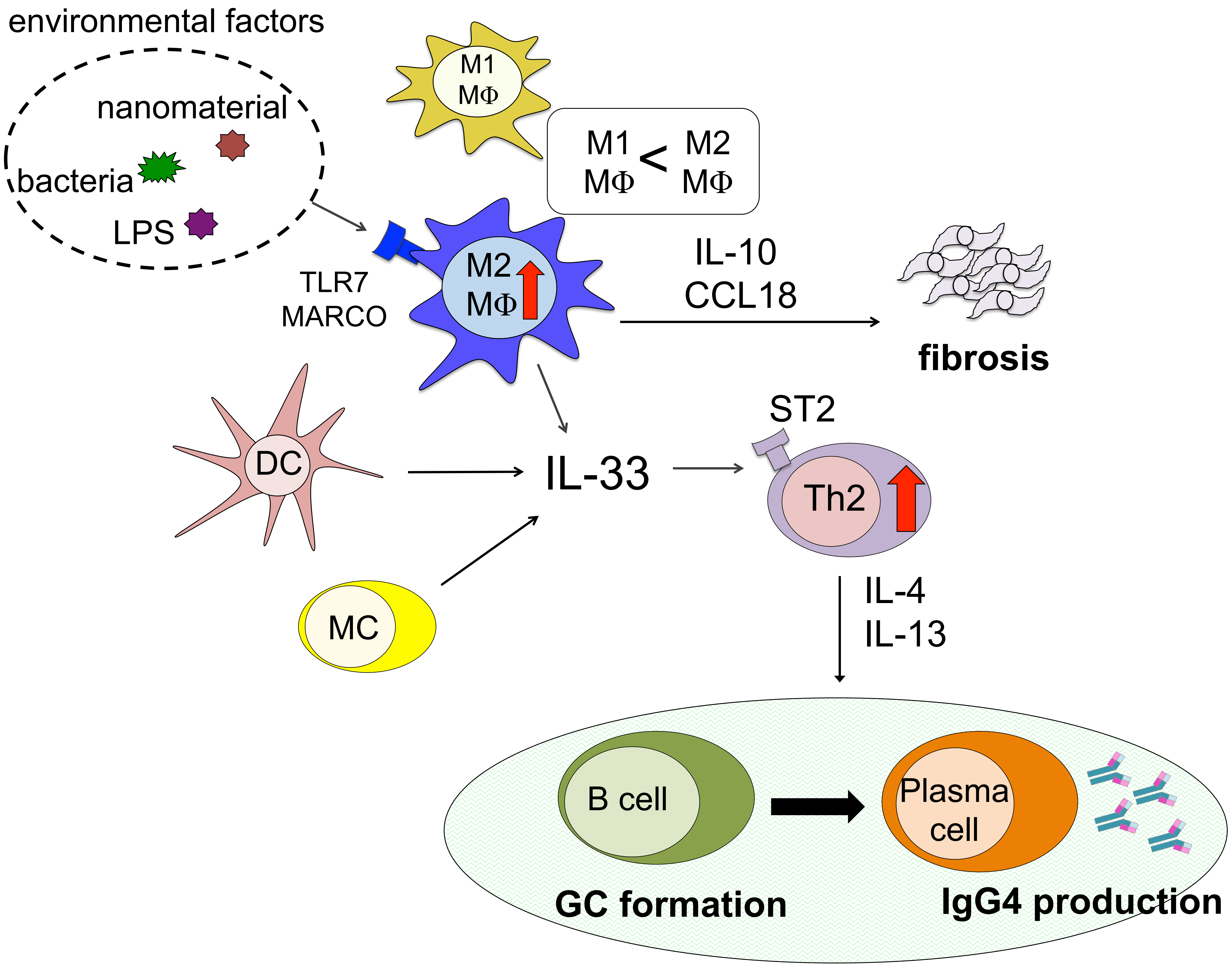

Supplementary Figure 3. Schematic model of IL-33 and innate immune network in IgG4-RD. M2 macrophages recognize some environmental factors and promote local fibrosis and production of T helper type 2 (Th2) cytokines via IL-33. Abbreviations: DC, dendritic cell; GC, germinal center; ; LPS, lipopolysaccharide; MARCO, macrophage receptor with collagenous structure; MC, mast cell; MΦ, macrophage; TLR, Toll-like receptor.
